# Supplementary material for: Schizophrenia, autism spectrum disorders and developmental disorders share specific disruptive coding mutations
Source: Nat Commun. 2021 Sep 9;12:5353. doi: 10.1038/s41467-021-25532-4 (PMC8429694; doi:10.1038/s41467-021-25532-4)
Supplement: Supplementary file 1 — Supplementary information [file 41467_2021_25532_MOESM1_ESM.pdf]

Supplementary Information

SZ DNV enrichment (Dependent variable)	Genes tested	Predictors	Estimate	Std. Error	P
SZ PTVs	13,015	NDD PTV P value	0.15	0.023	3.30E-11
		NDD miss P value	-0.018	0.041	0.67
SZ Missense	13,015	NDD PTV P value	0.012	0.014	0.38
		NDD miss P value	0.049	0.017	0.0047

Supplementary Table 1. Genome-wide analysis of mutation congruence in pleiotropic genes. A multivariable Poisson regression model was used to evaluate whether individual gene enrichment statistics in neurodevelopmental disorders predicts de novo variant enrichment in schizophrenia. This analysis tested 13,015 genes for which independent PTV and missense enrichment statistics were reported in the Deciphering Development Disorders study (Kaplanis et al 2020). SZ = schizophrenia; NDD PTV P value = per-gene enrichment P value for PTVs in developmental disorders; NDD miss P value = per-gene enrichment P value for missense variants in developmental disorders.

SZ DNV enrichment (Dependent variable)	Genes tested	Predictors	Estimate	Std. Error	P
SZ PTVs	12893	NDD PTV P value	0.14	0.023	1.20E-09
		NDD Miss P value	-0.034	0.042	0.41
		Brain_exp	0.069	0.035	0.053
		PTV o/e	-0.36	0.22	0.11
SZ missense	12991	NDD PTV P value	0.012	0.014	0.38
		NDD Miss P value	0.048	0.018	0.0069
		Brain_exp	0.021	0.014	0.14
		Miss o/e	0.11	0.13	0.41

Supplementary Table 2. Genome-wide analysis of mutation congruence in pleiotropic genes including gene brain expression and gene constraint scores as covariates. A multivariable Poisson regression model was used to evaluate whether individual gene enrichment statistics in neurodevelopmental disorders predicts de novo variant enrichment in schizophrenia. For per-gene measures of constraint, the gnomAD observed / expected constraint score was used (Karczewski et al 2020). Levels of gene expression in brain tissue were obtained from the BrainSeq project and defined as the level of expression in dorsolateral prefrontal cortex averaged over all available timepoints (second trimester to 85 years) (Jaffe et al 2018). SZ = schizophrenia; NDD PTV P value = per-gene enrichment P value for PTVs in developmental disorders; NDD miss P value = per-gene enrichment P value for missense variants in developmental disorders. PTV obs/exp = observed / expected number of protein-truncating variants per-gene in the gnomAD database. Miss obs/exp = observed / expected number of missense variants per-gene in the gnomAD database.

Varaint class	SZ DNVs in primary set		SZ DNVs outside primary set		P	Rate ratio (95% CI)
	**Observed**	**Expected**	**Observed**	**Expected**		
PTVs in LoF intolerant genes and missense variants (MPC ≥ 2)	9	1.2	202	186.1	1.10E-05	6.91 (3.11, 13.38)

Supplementary Table 3. Enrichment of schizophrenia de novo variants in the primary set after conditioning on the background schizophrenia de novo rate. A two-sample Poisson rate ratio test is used to compare the enrichment of schizophrenia de novo variants in the primary set to the enrichment of all schizophrenia PTVs in LoF intolerant genes and missense variants with MPC scores ≥ 2. P value is uncorrected and two-tailed.

NDD variant set	N variants tested	Schizophrenia *de novo* variant enrichment		
		Observed / expected	P	Rate Ratio (95% CI)
PTVs in primary set	2,118	3 / 0.44	0.01	6.77 (1.40, 19.80)
Missense variants in primary set	3,745	6 / 0.76	0.00014	7.90 (2.90, 17.19)

Supplementary Table 4. Enrichment tests of schizophrenia de novo PTVs and missense variants in the NDD primary variant set. The number of observed and expected *de novo* variants from the neurodevelopmental variant sets in 3,444 schizophrenia trios is shown. Enrichment statistics were generated using a Poisson rate ratio test. P-values are uncorrected and two-tailed. PTVs = Protein-truncating variants; NDD = neurodevelopmental disorders; CI = confidence interval.

NDD variant set	N variants tested	Schizophrenia case-control analysis					
		Case variants	Control variants	Case rate	Control rate	P	Odds ratio (95% CI)
PTVs in primary set	4,199	9	4	0.0022	0.0007	0.024	2.99 (1.01, 10.3)
Missense variants in primary set	3,777	9	9	0.0022	0.0016	0.25	1.36 (0.54, 3.44)

Supplementary Table 5. Schizophrenia case-control analysis of PTVs and missense variants in the NDD primary variant set. Firth's penalised logistic regression models is used to evaluate the burden of NDD variants in 4,070 schizophrenia cases and 5,712 controls. P-values are uncorrected and one-tailed.

Gene	Variant	Decipher Patient ID	Decipher Pathogenicity	Decipher Inheritance	Decipher Phenotypes
AUTS2	7:69364416:C:T	290793	Likely pathogenic	De novo	Abnormal emotion/affect behavior, Abnormal facial shape, Hyperactivity, Moderate global developmental delay
CSNK2A1	20:472926:T:C	265664	Pathogenic	De novo	Abnormal pattern of respiration, Brachydactyly, Global developmental delay, Leukonychia, Thick lower lip vermilion, Wide mouth
CSNK2A1	20:472926:T:C	280954	Likely pathogenic	De novo	Broad face, Broad nasal tip, Downturned corners of mouth, Global developmental delay, Joint hypermobility, Postaxial polydactyly, Sparse scalp hair
NF1	17:29679366:C:T	275376	Pathogenic	De novo	Abnormality of prenatal development or birth, Abnormality of skin pigmentation, Abnormality of the abdominal wall, Abnormality of the intestine, Delayed gross motor development, Delayed speech and language development, Epicanthus, Global developmental delay, Neurofibromas, Specific learning disability
KMT2D	12:49420670:G:A	397870	Pathogenic	De novo	No phenotypes have been entered into DECIPHER for this patient.

Supplementary Table 6. Phenotypes from DECIPHER patients who carry a variant from the NDD primary variant set that is also observed as a de novo mutation in schizophrenia.
