# Supplementary material for: Schizophrenia, autism spectrum disorders and developmental disorders share specific disruptive coding mutations
Source: Nat Commun. 2021 Sep 9;12:5353. doi: 10.1038/s41467-021-25532-4 (PMC8429694; doi:10.1038/s41467-021-25532-4)
Supplement: Supplementary file 3 — Description of Additional Supplementary Files [file 41467_2021_25532_MOESM3_ESM.pdf]

Description of additional supplementary files

File name: Supplementary Data 1

Description: List of de novo variants observed in both schizophrenia and neurodevelopmental disorder trios. 'NDD phenotype' indicates whether the variant was reported in a developmental disorder (DD) trio or an autism spectrum disorder (ASD) trio. Asterisks indicate genes associated at exome-wide significance with DNVs in NDDs (Kaplanis et al 2020). The 'NDD variant set' column indicates whether the variant observed in both schizophrenia and neurodevelopmental disorder trios was included in our primary or comparator NDD variant set, and the parentheses show the total number of NDD variants within the set. Chr = chromosome; pos = genomic position (in build 37); ref = reference allele; alt = alternative allele; MPC = "Missense badness, Polyphen-2, Constraint" pathogenicity score; pLi = "probability of loss-of-function intolerance".

File name: Supplementary Data 2

Description: Schizophrenia case-control variants observed in the NDD primary variant set. Variant frequencies in the gnomAD data set (separately for both the exome data and all samples without a neurological phenotypes) are provided. For variants observed in the ClinVar database, the recorded ClinVar phenotypes and pathogenicity rank are provided.

File name: Supplementary Data 3

Description: Schizophrenia de novo variants observed in the NDD primary variant set. Variant frequencies in the Gnomad non-neuro data set (samples without a neurological phenotype) are provided. For variants observed in the ClinVar database, the recorded ClinVar phenotypes and pathogenicity rank are provided. Where available, additional phenotypes and age-of-onset for probands carrying a primary variant were obtained from the original publications. For variants observed in the DECIPHER database, their recorded pathogenicity status is provided.

File name: Supplementary Data 4

Description: Functions and conditions associated with genes affected by the primary neurodevelopmental disorder variants observed as schizophrenia de novo variants. Recurrent Human Phenotype Ontology (HPO) terms are described for development disorder patients who carry mutations within CSNK2A1, SCN2A, AUTS2 and SLC6A1; this data was taken from the 2017 Deciphering Developmental Disorders study.

File name: Supplementary Data 5

Description: Studies used to obtain schizophrenia de novo variants. Sample descriptions, ascertainment details and diagnostic criteria were taken from the original publications.

File name: Supplementary Data 6

Description: Neurodevelopmental disorder gene sets tested in genic pleiotropy analysis. Neurodevelopmental disorder associated genes were identified from the Deciphering Developmental Disorders study (Kaplanis et al. 2020). Neurodevelopmental disorder associated genes were defined as those enriched for a given class of de novo variant (PTVs or missense variants) in developmental disorders with a P value $< 2.5 \times 10^{-6}$ (i.e. Bonferroni correction for 20,000 genes). Unadjusted DD PTV and missense P values were taken from Kaplanis et al 2020, which were generated using the DeNovoWest method.

File name: Supplementary Data 7

Description: Neurodevelopmental disorder variants tested in the allelic pleiotropy analysis. Neurodevelopmental disorder variants are defined as de novo variants observed in the largest autism spectrum disorder and developmental disorder proband-parent sequencing studies ((Kaplanis et al. 2020; Satterstrom et al. 2020). Variants are stratified into two sets: 1) The primary variant set contains PTVs in loss-of-function intolerant genes (genes with gnomAD pLi scores ≥ 0.9 (Karczewski et al. 2020)) and missense variants with MPC scores

≥ 2 (Samocha et al. 2017). The comparator variant set contained all remaining variants (PTVs in genes with pLi scores < 0.9, missense variants with MPC scores < 2 and all synonymous variants). The number of schizophrenia de novo variants expected to occur in the primary or negative control set was estimated by summing the tri-nucleotide mutation rates for all variants in the given set.
