# Supplementary material for: Schizophrenia, autism spectrum disorders and developmental disorders share specific disruptive coding mutations
Source: Nat Commun. 2021 Sep 9;12:5353. doi: 10.1038/s41467-021-25532-4 (PMC8429694; doi:10.1038/s41467-021-25532-4)
Supplement: Supplementary file 7 — Supplementary Data 4 [file 41467_2021_25532_MOESM7_ESM.docx]

**Supplementary Data 4**. Functions and conditions associated with genes affected by the primary neurodevelopmental disorder variants observed as schizophrenia de novo variants. Recurrent Human Phenotype Ontology (HPO) terms are described for development disorder patients who carry mutations within *CSNK2A1*, *SCN2A*, *AUTS2* and *SLC6A1*; this data was taken from the 2017 Deciphering Developmental Disorders study.

| **Gene ID** | **Gene Name** | ***Alternative ID*** | **General Function** | **Related Conditions** | **Recurrent HPO terms in developmental disorder patients** | **Expression Profile** | **Key References** | **Notes** |
| --- | --- | --- | --- | --- | --- | --- | --- | --- |
| *KMT2D* | Lysine methyltransferase 2D | *ALR, MLL2, MLL4* | A methyltransferase, responsible for a majority of the H3K4me1/2 marks in mammalian cells along with KMT2C.  Together they have been linked to a role in neuronal differentiation.  KMT2D plays critical roles in regulating development, differentiation, metabolism, and tumour suppression. | Kabuki syndrome ( Autosomal Dominant) | Not available. | Ubiquitous expression across most bodily tissues, including brain (RPKM 3.2).  Important for embryonic development. | ^1–9^ | The encoded protein is part of a large protein complex called ASCOM.  ASCOM is a transcriptional regulator of the beta-globin and oestrogen receptor genes  Deficiency compromises the development of regulatory T cells (T-reg cells).  **N.B.** The similar gene KMT2F (also known as SET1, SETD1A) linked to SZ.  **N.B.B.** *KMT2C* has been implicated in Kleefstra syndrome.  ***N.B.B.B.*** *Kmt2a/Mixed-lineage leukemia 1* (*Mll1*), in mouse postnatal forebrain and adult prefrontal cortex (PFC) is associated with increased anxiety and cognitive deficits. |
| *NF1* | Neurofibromin 1 | *NFNS, VRNF, WSS* | Functions as a negative regulator of the Ras signal transduction pathway by stimulating GTPase activity of Ras. | Neurofibromatosis 1 – characterised by tumours of nerves and skin (neurofibromas),  Learning disabilities  Autosomal Dominant | Not available. | Ubiquitous expression across most bodily tissues, highly expressed in brain (RPKM 8.2) and thyroid (RPKM 9.3).  **N.B.** Homozygous LOF mutation unviable in mice. | ^10–14^ | Over 50% of individuals with NF1 experience learning disabilities. |
| *AUTS2* | Activator of transcription and developmental regulator | *FBRSL2, MRD26* | Component of a Polycomb group (PcG) multiprotein PRC1-like complex.  The PcG PRC1 complex mediates transcriptional activation of CNS genes via epigenetic remodelling of histones and chromatin.  AUTS2 regulates neuritogenesis via activation of Rac1 signalling and is involved in neural migration during embryo development. | Autism Spectrum Disorder (ASD), Schizophrenia  Autosomal Dominant | Global developmental delay; Abnormality of the brain; Microcephaly; Abnormal eye physiology; Abnormality of the mouth; Abnormality of the pinna; Abnormality of the hair; Abnormality of the foot; Abnormality of the eyelid | Ubiquitous expression across most bodily tissues including brain (RPKM: 1.95) brain.  **N.B.** Homozygous LOF mutation unviable in mice. | ^15–20^ | Implicated in neurodevelopment and as a candidate gene for numerous neurological disorders, including autism spectrum disorders, intellectual disability, and developmental delay. |
| *GRIA3* | Glutamate Ionotropic Receptor AMPA Type Subunit 3 | *GLUR3, GLURC, MRX94* | Glutamate receptor that functions as ligand-gated ion channel in the central nervous system. It plays an important role in excitatory synaptic transmission.  Codes for AMPA receptor subunit.  Mutations result in absent or diminished GluA3 protein. | Mental retardation (X-linked, Syndromic, wu type),  Intellectual Disability,  ASD, Major Depressive Disorder (sleep disturbances),  Methamphetamine Dependence (& associated psychosis)  Deletion or duplication can cause ID. | Not available. | Specific expression in brain (RPKM: 19.9), adrenal: (RPKM 3.0).  Important in embryonic brain development. | ^21–27^ | These receptors are heteromeric protein complexes composed of multiple subunits, arranged to form ligand-gated ion channels and are activated in a variety of normal neurophysiologic processes.  Increased levels of dopamine in the striatum of *Gria3KO* mice  **N.B.** Previously considered a BPD candidate gene. |
| *RUNX3* | Runt-related transcription factor 3 | *AML2 , CBFA3, PEBP2aC* | Forms the heterodimeric complex core-binding factor (CBF) with CBFB.  Regulator of CD8+ T-cell thymocyte development. | Cancer metastasis,  Pre-eclampsia,  Placental Dysfunction | Not available. | Biased expression in bone marrow (RPKM: 19.5) and spleen (RPKM16.7)  **N.B.** Homozygous LOF mutants unviable in mice. | ^28,29^ | Under epigenetic regulation. |
| *SLC6A1* | Voltage-dependent c-aminobutyric acid (GABA) transporter 1 (GAT-1) protein | *GABATHG, GABATR, GAT1 MAE* | Terminates the action of GABA by its high affinity sodium-dependent reuptake into presynaptic terminals. | Schizophrenia, ADHD,  Epileptic encephalopathy | Cognitive impairment; Abnormal eye physiology; Expressive language delay; Functional abnormality of the middle ear; Intellectual disability, moderate; Moderate global developmental delay; Hearing impairment; Abnormality of the mouth; Abnormality of finger. | SLC6A1 is primarily expressed in the adult brain (RPKM: 26.6), specifically, in GABAergic neurons and astrocytes.  Essential for embryonic development. | ^30–34^ | ﻿Mutations often result in loss of function of GAT-1 and thus reduced GABA re-uptake from the synapse. |
| *CSNK2A1* | Casein Kinase 2 Alpha 1 | *CK2A1, Cka1,*  *Cka2* | Casein kinase II is a serine/threonine protein kinase that phosphorylates acidic proteins such as casein. It is involved in various cellular processes, including cell cycle control, apoptosis, and circadian rhythm. | Neurodevelopmental disorders (Okur-Chung Neurodevelopmental Syndrome),  Intellectual disability,  Seizures | Global developmental delay; Abnormality of the nose; Broad nasal tip; Polydactyly; Depressed nasal bridge; Intellectual disability, severe; Epicanthus; Abnormality of the teeth; Joint hypermobility; Abnormality of calvarial morphology; Microcephaly; Abnormality of skin adnexa. | Ubiquitous expression across adult tissues including brain (RPKM: 25.8)  Essential for embryonic development. | ^35–38^ | Often associated dysmorphic facial features. ﻿Congenital heart abnormalities identified in nearly 30% of the patients with *CSNK2A1* mutations. |
| *KLHL20* | Kelch Like Family Member 20 | *KLEIP, KLHLX, KHLHX* | KLHL20 is a related CUL3-dependent ubiquitin ligase linked to autophagy, cancer, and Alzheimer's disease that promotes the ubiquitination and degradation of substrates including DAPK1, PML, and ULK1. | Alzheimer’s disease,  Cancer | Not available. | Ubiquitous expression across adult tissues including brain (RPKM: 4.7).  Important in Embryonic development. | ^39–42^ | ﻿Stress response of KLHL20 is linked to neurodegeneration. KLHL20 RNA transcript levels being among the top 20 biomarkers for Alzheimer’s disease progression. |
| *SCN2A* | Sodium Voltage-Gated Channel Alpha Subunit 2 | *BFIC3, BFIS3, BFNIS* | Mediates the voltage-dependent sodium ion permeability of excitable membranes.  Implicated in the regulation of hippocampal replay occurring within sharp wave ripples (SPW-R) important for memory | Autism Spectrum Disorder (ASD) | Seizures; Cognitive impairment; Abnormality of the lower limb; Abnormality of the teeth; Microcephaly. | Biased expression in brain (RPKM: 20.5). | ^43–45^ | Variants with these conditions are usually truncating, suggesting haploinsufficiency plays a major role. |

Key references from Supplementary Data 4

1. Shen, E., Shulha, H., Weng, Z. & Akbarian, S. Regulation of histone H3K4 methylation in brain development and disease. *Philosophical Transactions of the Royal Society B: Biological Sciences* **369**, (2014).

2. Froimchuk, E., Jang, Y. & Ge, K. Histone H3 lysine 4 methyltransferase KMT2D. *Gene* **627**, 337–342 (2017).

3. Vallianatos, C. N. & Iwase, S. Disrupted intricacy of histone H3K4 methylation in neurodevelopmental disorders. *Epigenomics* **7**, 503–518 (2015).

4. Placek, K. *et al.* MLL4 prepares the enhancer landscape for Foxp3 induction via chromatin looping. *Nat. Immunol.* **18**, 1035–1045 (2017).

5. Bon, van B. *et al.* MLL2 mutation detection in 86 patients with Kabuki syndrome: a genotype-phenotype study mutation detection in 86 patients with Kabuki syndrome: a genotype-phenotype study. Nothing to declare. *Clin Genet* **84**, 539–545 (2013).

6. Caciolo, C. *et al.* Neurobehavioral features in individuals with Kabuki syndrome. *Mol. Genet. Genomic Med.* **6**, 322–331 (2018).

7. Bjornsson, H. T. *et al.* Histone deacetylase inhibition rescues structural and functional brain deficits in a mouse model of Kabuki syndrome. *Sci. Transl. Med.* **6**, (2014).

8. Yamamoto, P. K. *et al.* Genetic and behavioral characterization of a *Kmt2d* mouse mutant, a new model for Kabuki Syndrome. *Genes, Brain Behav.* **18**, (2019).

9. Jakovcevski, M. *et al.* Neuronal Kmt2a/Mll1 histone methyltransferase is essential for prefrontal synaptic plasticity and working memory. *J. Neurosci.* **35**, 5097–5108 (2015).

10. Zhu, Y. *et al.* Ablation of NF1 function in neurons induces abnormal development of cerebral cortex and reactive gliosis in the brainZhu, Y., Romero, M. I., Ghosh, P., Ye, Z., Charnay, P., Rushing, E. J., … Parada, L. F. (2001). Ablation of NF1 function in neurons induce. *Genes Dev.* **15**, 859–876 (2001).

11. Cleven, A. H. G. *et al.* Loss of H3K27 tri-methylation is a diagnostic marker for malignant peripheral nerve sheath tumors and an indicator for an inferior survival. *Mod. Pathol.* **29**, 582–590 (2016).

12. Kehrer-Sawatzki, H., Mautner, V. F. & Cooper, D. N. Emerging genotype–phenotype relationships in patients with large NF1 deletions. *Human Genetics* **136**, 349–376 (2017).

13. De Raedt, T. *et al.* Somatic loss of wild type NF1 allele in neurofibromas: Comparison of NF1 microdeletion and non-microdeletion patients. *Genes Chromosom. Cancer* **45**, 893–904 (2006).

14. Bartelt-Kirbach, B., Wuepping, M., Dodrimont-Lattke, M. & Kaufmann, D. Expression analysis of genes lying in the NF1 microdeletion interval points to four candidate modifiers for neurofibroma formation. *Neurogenetics* **10**, 79–85 (2009).

15. Gao, Z. *et al.* An AUTS2-Polycomb complex activates gene expression in the CNS. *Nature* **516**, 349–354 (2014).

16. Oksenberg, N., Stevison, L., Wall, J. D. & Ahituv, N. Function and Regulation of AUTS2, a Gene Implicated in Autism and Human Evolution. *PLoS Genet.* **9**, e1003221 (2013).

17. McCarthy, S. E. *et al.* De novo mutations in schizophrenia implicate chromatin remodeling and support a genetic overlap with autism and intellectual disability. *Mol. Psychiatry* **19**, 652–658 (2014).

18. Zhang, B. *et al.* Association study identifying a new susceptibility gene (AUTS2) for Schizophrenia. *Int. J. Mol. Sci.* **15**, 19406–19416 (2014).

19. Hori, K. & Hoshino, M. Neuronal Migration and AUTS2 Syndrome. *Brain Sci.* **7**, (2017).

20. Bedogni, F. *et al.* Autism susceptibility candidate 2 (Auts2) encodes a nuclear protein expressed in developing brain regions implicated in autism neuropathology. *Gene Expr. Patterns* **10**, 9–15 (2010).

21. Iamjan, S. A., Thanoi, S., Watiktinkorn, P., Reynolds, G. P. & Nudmamud-Thanoi, S. Genetic variation of GRIA3 gene is associated with vulnerability to methamphetamine dependence and its associated psychosis. *J. Psychopharmacol.* **32**, 309–315 (2018).

22. Davies, B. *et al.* A point mutation in the ion conduction pore of AMPA receptor GRIA3 causes dramatically perturbed sleep patterns as well as intellectual disability. *Hum. Mol. Genet.* **26**, 3869–3882 (2017).

23. Bai, Z. & Kong, X. X-linked mental retardation combined with autism caused by a novel hemizygous mutation of GRIA3 gene. *Zhonghua Yi Xue Yi Chuan Xue Za Zhi* **36**, 829–833 (2019).

24. Philippe, A. *et al.* Xq25 duplications encompassing GRIA3 and STAG2 genes in two families convey recognizable X-linked intellectual disability with distinctive facial appearance. *Am. J. Med. Genet. Part A* **161**, 1370–1375 (2013).

25. Soto, D., Altafaj, X., Sindreu, C. & Bayés, À. Glutamate receptor mutations in psychiatric and neurodevelopmental disorders. *Communicative and Integrative Biology* **7**, e27887-1-e27887-6 (2014).

26. Gécz, J. *et al.* Characterization of the human glutamate receptor subunit 3 gene (GRIA3), a candidate for bipolar disorder and nonspecific X-linked mental retardation. *Genomics* **62**, 356–368 (1999).

27. Moretto, E., Passafaro, M. & Bassani, S. X-Linked ASDs and ID Gene Mutations. in *Neuronal and Synaptic Dysfunction in Autism Spectrum Disorder and Intellectual Disability* 129–150 (Elsevier Inc., 2016). doi:10.1016/B978-0-12-800109-7.00009-1

28. Zhang, Y. *et al.* Association between RUNX3 gene polymorphisms in severe preeclampsia and its clinical features. *Medicine (Baltimore).* **98**, e14954 (2019).

29. Manandhar, S. & Lee, Y. M. Emerging role of RUNX3 in the regulation of tumor microenvironment. *BMB Reports* **51**, 174–181 (2018).

30. Szatkiewicz, J. P. *et al.* Characterization of single gene copy number variants in schizophrenia. *Biol. Psychiatry* (2019). doi:10.1016/j.biopsych.2019.09.023

31. Rees, E. *et al.* De novo mutations identified by exome sequencing implicate rare missense variants in SLC6A1 in schizophrenia. *Nat. Neurosci.* **23**, 179–184 (2020).

32. Johannesen, K. M. *et al.* Defining the phenotypic spectrum of SLC6A1 mutations. *Epilepsia* **59**, 389–402 (2018).

33. Carvill, G. L. *et al.* Mutations in the GABA transporter SLC6A1 cause epilepsy with myoclonic-atonic seizures. *Am. J. Hum. Genet.* **96**, 808–815 (2015).

34. Yuan, F. fen, Gu, X., Huang, X., Zhong, Y. & Wu, J. SLC6A1 gene involvement in susceptibility to attention-deficit/hyperactivity disorder: A case-control study and gene-environment interaction. *Prog. Neuro-Psychopharmacology Biol. Psychiatry* **77**, 202–208 (2017).

35. Owen, C. I. *et al.* Extending the phenotype associated with the *CSNK2A1-* related Okur-Chung syndrome-A clinical study of 11 individuals. *Am. J. Med. Genet. Part A* **176**, 1108–1114 (2018).

36. Nakashima, M. *et al.* Identification of de novo CSNK2A1 and CSNK2B variants in cases of global developmental delay with seizures. *J. Hum. Genet.* **64**, 313–322 (2019).

37. Okur, V. *et al.* De novo mutations in CSNK2A1 are associated with neurodevelopmental abnormalities and dysmorphic features. *Hum. Genet.* **135**, 699–705 (2016).

38. Poirier, K. *et al.* CSNK2B splice site mutations in patients cause intellectual disability with or without myoclonic epilepsy. *Hum. Mutat.* **38**, 932–941 (2017).

39. Liu, C. C. *et al.* Cul3-KLHL20 Ubiquitin Ligase Governs the Turnover of ULK1 and VPS34 Complexes to Control Autophagy Termination. *Mol. Cell* **61**, 84–97 (2016).

40. Feng, Y. & Klionsky, D. J. Downregulation of autophagy through CUL3-KLHL20-mediated turnover of the ULK1 and PIK3C3/VPS34 complexes. *Autophagy* **12**, 1071–1072 (2016).

41. Chen, Z., Picaud, S., Filippakopoulos, P., D’Angiolella, V. & Bullock, A. N. Structural Basis for Recruitment of DAPK1 to the KLHL20 E3 Ligase. *Structure* **27**, 1395-1404.e4 (2019).

42. Arefin, A. S., Mathieson, L., Johnstone, D., Berretta, R. & Moscato, P. Unveiling Clusters of RNA Transcript Pairs Associated with Markers of Alzheimer’s Disease Progression. *PLoS One* **7**, e45535 (2012).

43. Spratt, P. W. E. *et al.* The Autism-Associated Gene Scn2a Contributes to Dendritic Excitability and Synaptic Function in the Prefrontal Cortex. *Neuron* **103**, 673-685.e5 (2019).

44. Sanders, S. J. *et al.* Progress in Understanding and Treating SCN2A-Mediated Disorders. *Trends in Neurosciences* **41**, 442–456 (2018).

45. Wolff, M., Brunklaus, A. & Zuberi, S. M. Phenotypic spectrum and genetics of SCN2A-related disorders, treatment options, and outcomes in epilepsy and beyond. *Epilepsia* **60**, S59–S67 (2019).
