# Supplementary material for: Schizophrenia, autism spectrum disorders and developmental disorders share specific disruptive coding mutations
Source: Nat Commun. 2021 Sep 9;12:5353. doi: 10.1038/s41467-021-25532-4 (PMC8429694; doi:10.1038/s41467-021-25532-4)
Supplement: Supplementary file 11 — Reporting Summary [file 41467_2021_25532_MOESM11_ESM.pdf]

nature research

Corresponding author(s): Michael O'Donovan and Michael Owen

Last updated by author(s): Jul 9, 2021

Reporting Summary

Nature Research wishes to improve the reproducibility of the work that we publish. This form provides structure for consistency and transparency in reporting. For further information on Nature Research policies, see our Editorial Policies and the Editorial Policy Checklist.

Statistics

For all statistical analyses, confirm that the following items are present in the figure legend, table legend, main text, or Methods section.

n/a	Confirmed	
☐	☒	The exact sample size (*n*) for each experimental group/condition, given as a discrete number and unit of measurement
☒	☐	A statement on whether measurements were taken from distinct samples or whether the same sample was measured repeatedly
☐	☒	The statistical test(s) used AND whether they are one- or two-sided<br>*Only common tests should be described solely by name; describe more complex techniques in the Methods section.*
☒	☐	A description of all covariates tested
☐	☒	A description of any assumptions or corrections, such as tests of normality and adjustment for multiple comparisons
☐	☒	A full description of the statistical parameters including central tendency (e.g. means) or other basic estimates (e.g. regression coefficient) AND variation (e.g. standard deviation) or associated estimates of uncertainty (e.g. confidence intervals)
☐	☒	For null hypothesis testing, the test statistic (e.g. F, t, r) with confidence intervals, effect sizes, degrees of freedom and P value noted<br>*Give P values as exact values whenever suitable.*
☒	☐	For Bayesian analysis, information on the choice of priors and Markov chain Monte Carlo settings
☒	☐	For hierarchical and complex designs, identification of the appropriate level for tests and full reporting of outcomes
☐	☒	Estimates of effect sizes (e.g. Cohen's *d*, Pearson's *r*), indicating how they were calculated

Our web collection on statistics for biologists contains articles on many of the points above.

Software and code

Policy information about availability of computer code

Data collection	No software was used for data collection.
Data analysis	Statistical analyses were conducted in R software (v 3.4.3). Standard R packages were used to perform poisson rate tests (poisson.test() function). Ensemble Variant Effect Predictor (version 96) was used to annotate schizophrenia de novo variants. R code used for this research is available at https://github.com/reeseg/SZ_NDD_pleiotropy_analysis.

For manuscripts utilizing custom algorithms or software that are central to the research but not yet described in published literature, software must be made available to editors and reviewers. We strongly encourage code deposition in a community repository (e.g. GitHub). See the Nature Research guidelines for submitting code & software for further information.

Data

Policy information about availability of data

All manuscripts must include a data availability statement. This statement should provide the following information, where applicable:

- Accession codes, unique identifiers, or web links for publicly available datasets
- A list of figures that have associated raw data
- A description of any restrictions on data availability

All schizophrenia de novo variants were obtained from the following published sources outlined in Supplementary Data 5, and from the following DOIs: https://doi.org/10.1038/s41593-019-0565-2, https://doi.org/10.1038/nature12929, https://doi.org/10.1038/ng.886, https://doi.org/10.1038/ng.2446, https://doi.org/10.1016/j.cell.2013.06.049, https://doi.org/10.1038/srep18209, https://doi.org/10.1038/ejhg.2015.218, https://doi.org/10.1371/journal.pone.0112745, https://doi.org/10.1038/mp.2014.29, https://doi.org/10.1038/s41593-019-0564-3. De novo variants from ASD trios were obtained from Satterstrom et al 202021 (https://doi.org/10.1016/j.cell.2019.12.036), and de novo variants from DD trios were obtained from Kaplanis et al 202014 (https://doi.org/10.1038/s41586-020-2832-5). DD gene level association statistics were obtained from Kaplanis et al 202014 (https://doi.org/10.1038/s41586-020-2832-5).

The Swedish case control exome sequencing data set is available through dbGaP accession number phs000473.v2.p2.

Field-specific reporting

Please select the one below that is the best fit for your research. If you are not sure, read the appropriate sections before making your selection.

☒ Life sciences ☐ Behavioural & social sciences ☐ Ecological, evolutionary & environmental sciences

For a reference copy of the document with all sections, see nature.com/documents/nr-reporting-summary-flat.pdf

Life sciences study design

All studies must disclose on these points even when the disclosure is negative.

Sample size	No statistical methods were used to pre-determine sample sizes. Our schizophrenia trio sample consists of all publicly available data from exome-sequencing studies of de novo variants in schizophrenia.
Data exclusions	No data exclusions were required because all genetic data was obtained from published studies, where sample and variant quality control had already been performed. See Supplementary Data 5 for a list of these original studies.
Replication	We sought one independent replication experiment of de novo variant findings from our analysis of allelic pleiotropy using exome sequencing data from 4,070 schizophrenia cases and 5,712 controls. In this sample, we replicated the increased rate of NDD primary variants in schizophrenia (P = 0.036; odds ratio (95% CI) = 1.90 (0.94, 3.95).
Randomization	Randomization of experimental groups is not applicable to this study. Probands were allocated to the case group on the basis of having a DSM-IV or ICD-10 diagnosis of schizophrenia or schizoaffective disorder, apart from 5 probands who had a diagnosis of non-organic psychosis (details in Supplementary Data 5). To test the enrichment of de novo variants in a given gene, or across groups of variants observed in neurodevelopmental disorders, the observed de novo rate was compared to the expected de novo rate, which is based on known tri-nucleotide mutation rates, and thus does not require randomization.
Blinding	Proband-parent trios were ascertained blind to any genome analysis.

Reporting for specific materials, systems and methods

We require information from authors about some types of materials, experimental systems and methods used in many studies. Here, indicate whether each material, system or method listed is relevant to your study. If you are not sure if a list item applies to your research, read the appropriate section before selecting a response.

Materials & experimental systems

n/a	Involved in the study	
☒	☐	Antibodies
☒	☐	Eukaryotic cell lines
☒	☐	Palaeontology and archaeology
☒	☐	Animals and other organisms
☐	☒	Human research participants
☒	☐	Clinical data
☒	☐	Dual use research of concern

Methods

n/a	Involved in the study	
☒	☐	ChIP-seq
☒	☐	Flow cytometry
☒	☐	MRI-based neuroimaging

Human research participants

Policy information about studies involving human research participants

Population characteristics	All probands included in the current study had received a DSM-IV or ICD-10 diagnosis of schizophrenia, apart from 5 probands who had a diagnosis of non-organic psychosis (details in Supplementary Data 5). As we used a de novo variant study design, our results are controlled for population biases
Recruitment	No new participants were recruited for the current study. All schizophrenia de novo variant data was obtained from the publishes sources outlined in Supplementary Data 5. These probands were ascertained from psychiatric wards or outpatient clinics, and all had received a DSM-IV (Diagnostic and Statistical Manual of Mental Disorders; fourth edition) or ICD-10 (International Statistical Classification of Diseases and Related Health Problems; 10th revision) research diagnosis of schizophrenia or schizoaffective disorder, apart from 5 probands who had a diagnosis of non-organic psychosis.
Ethics oversight	Research Ethics Committee for Wales

Note that full information on the approval of the study protocol must also be provided in the manuscript.
